# Xiaotan Jieyu Prescription Alleviates Breast Precancerous Lesions through PI3K/Akt Signaling Pathway

**DOI:** 10.1155/2020/4129461

**Published:** 2020-08-12

**Authors:** Jing Zhao, Tao Pang, Jian-peng Jiao, Bin Wang, Xuan Liu, Li-juan Xiu, Da-zhi Sun, Xiao-qiang Yue, Chao-qin Yu

**Affiliations:** ^1^Department of Traditional Chinese Medicine, Changzheng Hospital, Navy Medical University, Shanghai 200003, China; ^2^Department of Pharmacy, Changzheng Hospital, Navy Medical University, Shanghai 200003, China; ^3^Department of General Surgery (III), Changzheng Hospital, Navy Medical University, Shanghai 200003, China; ^4^Department of Traditional Chinese Medicine Gynecology, Changhai Hospital, Navy Medical University, Shanghai 200433, China

## Abstract

**Methods:**

The successfully established breast precancerous lesion rat model and normal healthy rats were randomly assigned into the blank (BLA), model (MOD), XTJY-low (LD), XTJY-medium (MD), XTJY-high (HD), and tamoxifen (TAM) groups. Different concentrations of XTJY and saline were supplied by intragastric administration for 4 consecutive weeks to assess the protective effect of XTJY on the progress of the breast precancerous lesion in rats involving the phosphatidylinositol-3-kinase (PI3K)/protein kinase B (Akt) signaling pathway.

**Results:**

In this study, it determined that 10 mg/each rat DMBA-combined estrogen and progesterone induction for 10 weeks was the optimal condition for the establishment of the breast precancerous lesion rat model. In vivo administration of XTJY or TAM was found to inhibit the development of the breast precancerous lesion, and the occurrence rate of breast invasive carcinomas was decreased by about 50%. Furthermore, XTJY or TAM markedly reduced protein expressions of PI3K and p-Akt and increased protein expressions of PTEN.

**Conclusion:**

These data indicated that XTJY can significantly alleviate the development of breast precancerous lesions by inhibiting the activation of the PI3K/Akt signaling pathway. XTJY may be a promising drug for the treatment of precancerous lesions in breast cancer.

## 1. Introduction

Breast cancer (BC) is the most common cancer in women, accounting for approximately 29% of female malignancy, and is the leading cause of cancer-related death in women [1,2]. A previous study found that there are∼300,000 additional patients with BC in China each year [3]. At the end of the 20th century, researchers developed a “multistage development model for breast cancer,” which states that breast cancer had experienced the continuous process containing normal, hyperplasia, atypical hyperplasia, and carcinoma [4]. Before becoming invasive carcinomas, the malignant transformation is reversible and can be blocked or reversed by specific drug intervention. Therefore, the incidence of breast cancer can be actually reduced by blocking the tumor development process.

Precancerous lesions are diseases that have greater than 20% risk of developing into cancer; notably, breast precancerous lesions mainly include atypical ductal hyperplasia (ADH), ductal carcinoma in situ (DCIS), ductal carcinoma, and papillary tumor, which resulted in approximately 50% probability of breast cancer occurrence [5]. Currently, treatment methods for breast precancerous lesions mainly include surgical resection and drug therapy, but both of them are controversial. In modern medicine, tamoxifen is widely used to prevent and treat benign and malignant breast lesions as a selective estrogen receptor antagonist, but tamoxifen may also affect the risk factors of cardiac and thromboembolism disease [6]. Therefore, there is still a lack of effective means to treat breast precancerous lesions.

Traditional Chinese medicine (TCM) had been extensively used in China and proved effective in the prevention and treatment of various diseases. “Xiaotan Jieyu (XTJY) prescription” is a TCM prescription established by professor Pin-Kang Wei after summarizing years of clinical experience. Clinical observation shows that XTJY can effectively treat breast precancerous lesions, reduce or even reverse the pathological state, and extend the survival time of patients. XTJY consisted of 12 traditional Chinese herbs, including rhizoma arisaematis, *Bupleurum*, fried *Atractylodes*, Hangbaishao, tangerine peel, *Pinellia ternate*, *Poria cocos*, *Angelica sinensis*, and Indian Iphigenia Bulb. Above with the use of herbs could invigorate the circulation of Qi and dissipate phlegm and resolve mass. At present, modern pharmacological research had shown that activating blood, removing phlegm, and dredging the liver to use qi medicine had their unique antitumor mechanism [7, 8]. Our previous study revealed that XTJY can inhibit the proliferation of human breast precancerous lesion MCF-10AT cells by inhibiting the PI3K/Akt signaling pathway [9]. However, there is no in vivo evidence to further verify that XTJY has an operative intervention effect on breast precancerous lesions.

In the present study, breast precancerous lesion model rats induced by DMBA-combined estrogen and progesterone were treated with different concentrations of XTJY to investigate the biological effects of XTJY on antibreast precancerous lesions. The data demonstrated that the incidence of premalignant breast disease was 91.7% induced by DMBA-combined estrogen and progesterone. Besides, it has been proved that XTJY significantly alleviated breast precancerous lesions of rats at least partially through the PI3K/Akt signaling pathway.

## 2. Materials and Methods

### 2.1. Reagents

7, 12-Dimethylbenz anthracene (DMBA; D0677) was obtained from Tokyo Chemical Industry Co., Ltd (Tokyo, Japan). Estradiol benzoate injection (lot number: B130902) was purchased from Ningbo Sansheng Pharmaceutical Co., Ltd. Progesterone injection (lot number: H31021401) was purchased from Shanghai General Pharmaceutical Co., Ltd. Tamoxifen (TAM, lot number: 140102) was purchased from Yangtze River Pharmaceutical Group (Jiangsu, China). Additional reagents employed in the present study were commercially available and of analytical purity.

### 2.2. Drug Preparation for Rat Models of Breast Precancerous Lesions

#### 2.2.1. Preparation of DMBA Solution

The DMBA was precisely weighed, dissolved in sesame oil according to the proportion of 10 mg/ml, placed in an ultrasonic constant temperature water bath box (60°C, 40 Hz), and completely dissolved by ultrasonic oscillation.

#### 2.2.2. Preparation of TAM Solution

Four tablets (10 mg^*∗*^4) of TAM were dissolved in 50 ml of normal saline and prepared into tamoxifen solution with a concentration of 0.8 mg/ml.

#### 2.2.3. Preparation of Xiaotan Jieyu (XTJY) Prescription

The extract of XTJY was made by Shanghai Zhangjiang Medicine Valley Public Service Platform Co., Ltd (Shanghai, China). XTJY was prepared according to the following procedures: these herbs were boiled for 2 h 8 times (v/w) with distilled water, then these herbs were boiled 2 times with distilled water again (each 1 h), and then the filtrate was collected and mixed with the previously collected filtrate and concentrated. The concentrate was precipitated 2 times (v: v) with ethyl alcohol for 48 h. The supernatant was collected and then further concentrated to 3.35 g/ml.

### 2.3. Animals

A total of 123 female Sprague Dawley rats (190–210g, 6 weeks old) were purchased from Shanghai SIPPR/BK Laboratory Animals Ltd., Shanghai, China (Production license: SCXK (Shanghai) 2013–0016). All animals were housed in polycarbonate cages in an animal laboratory with 12 h light/12 h dark cycles under conditions of controlled temperature 23 ± 2°C and in a humidified atmosphere (55 ± 15%). All experiments were carried out in accordance with the National Institutes of Health Guide for the Care and Use of Laboratory Animals (NIH Publications no. 8023, revised 1978). The experimental protocol was subject to approval by the Animal Ethics Committee of Second Military Medical University.

### 2.4. Establishment of the Breast Precancerous Lesion Rat Model

Following a week of adaptation, rats were randomly divided into four groups as follows: the blank (BLA, *n* = 15), DMBA-low (DLD, *n* = 36), DMBA-medium (DMD, *n* = 36), and DMBA-high (DHD, *n* = 36) groups. The breast precancerous lesion SD rat model was induced using DMBA in combination with estrogen and progesterone as described previously [10]. The rats in the DLD, DMD, and DHD groups received one-time gavage using 5 mg/each rat, 10 mg/each rat, and 20 mg/each rat DMBA sesame oil, respectively. The rats in the BLA group received one-time gavage using 2 ml/each rat saline and were conventionally fed for 12 cycles. The next five days acted as a cycle (5-day cycle), and 0.5 mg/kg estradiol benzoate injection was injected in the medial muscles of the hind legs from the first day to the third day. Then, 4 mg/kg progesterone injection was injected in the medial muscles of the hind legs from the fourth day. The rats were noted on the fifth day. Continuous 12 cycles (5-day cycle) were carried out. Following 8, 10, or 12 weeks, the mice in the BLA (*n* = 5), DLD (*n* = 12), DMD (*n* = 12), and DHD (*n* = 12) groups were sacrificed by cervical dislocation after being anesthetized by an intraperitoneal injection of 3% pentobarbital sodium (50 mg/kg).

### 2.5. Therapeutic Intervention Effect of XTJY Prescription on Breast Precancerous Lesion Model Rats

Rats were randomly divided into six groups as follows: the blank (BLA, *n* = 20), model (MOD, *n* = 20), XTJY-low (LD, *n* = 20), XTJY-medium (MD, *n* = 20), XTJY-high (HD, *n* = 20), and TAM (*n* = 20) groups. Treatment intervention was launched 11 weeks after the establishment of the rat model of the breast precancerous lesion. The rats in the LD, MD, and HD groups received 2 g/kg, 4 g/kg, and 8 g/kg XTJY prescription intragastrically. The rats in the TAM group received 8 mg/kg TAM by intragastric administration. The rats in the BLA and MOD groups received 10 ml/kg saline by intragastric administration. The treatment in each group was given once a day for 4 consecutive weeks. The rats were evaluated once a week, and their life characteristics were observed every day after treatment. Once treatment was complete, rats were sacrificed by cervical dislocation after being anesthetized by an intraperitoneal injection of 3% pentobarbital sodium (50 mg/kg) and tumors measured. The thymus and spleen tissues were collected and weighed for measuring the thymus index and spleen index. The final animal numbers for this research were *n* = 20 (BLA), *n* = 20 (MOD), *n* = 19 (LD), *n* = 19 (MD), *n* = 20 (HD), and *n* = 20 (TAM).

### 2.6. HE Staining and Immunohistochemistry Staining

Breast tissues from the rats were fixed in 4% paraformaldehyde at room temperature for 48–72 h. The breast tissue was embedded in paraffin, and 5 *μ*m-thick paraffin sections were prepared. The pathological changes of breast tissues were observed under the light microscope following hematoxylin and eosin (HE) staining. For IHC staining, the paraffin sections were deparaffinized with xylene and 3% hydrogen peroxide for antigen retrieval at room temperature for 10 min. Then, the sections were incubated with primary antibodies against PTEN (1 : 100; ab31392; Abcam, CA, USA), PI3K p85 (1 : 200; ab189403; Abcam), and p-Akt (1 : 50; ab38449; Abcam) at 4°C overnight followed by incubation with the appropriate amount of biotin-conjugated goat anti-rabbit IgG (BA1003) or biotin-conjugated goat anti-mouse IgG (BA1001; Wuhan Boster Biological Technology, Ltd., Wuhan, China) for 30 min at 37°C. Next, the reaction was visualized using AEC (Beijing Solarbio Science & Technology Co., Ltd., Beijing, China) and counterstained with hematoxylin. Positive immune staining was presented as brown or yellow granules in the cytoplasm or the nucleus.

### 2.7. Western Blot Assay

The protein samples were drawn from breast tissues of rats by using radioimmunoprecipitation assay lysis buffer (Wuhan Boster Biological Technology, Ltd). Protein was quantified with a BCA protein quantitative kit (Boster). The protein samples (20 *μ*g) were separated by 10% SDS-PAGE and then transferred to a PVDF (Millipore, USA) membrane. Following blocking with 5% BSA, the membrane was incubated with primary antibodies at 4°C overnight. The primary antibodies included rabbit anti-PTEN (1 : 1,000; ab31392; Abcam, CA, USA), mouse anti-PI3K p85 (1 : 1,000; ab189403; Abcam), rabbit anti-p-Akt (1 : 500; ab38449; Abcam), and mouse anti-*β*-actin (1 : 1,000; ab8226; Abcam). Then, the membranes were rinsed with TBST and incubated with HRP-conjugated goat anti-rabbit IgG (ab6721; 1 : 5,000; Abcam) or HRP-conjugated goat anti-mouse IgG (ab205719; 1 : 5,000; Abcam) at room temperature for 2 h. *β*-Actin was used as an inner loading control. The protein bands were identified by an ECL chemiluminescence kit (Millipore) and analyzed by Image-Pro Plus software.

### 2.8. Statistical Analysis

Statistical analysis was performed using SPSS19.0 software (IBM Corp., Armonk, NY, USA). The normal distribution data among multiple groups were compared by one-way analysis of variance (ANOVA), and differences between two groups were compared by the least significant difference. *P* values <0.05 were considered to indicate statistically significant differences.

## 3. Results

### 3.1. DMBA-Combined Estrogen and Progesterone Induced the Breast Precancerous Lesion Rat Model

**Figure 1:**
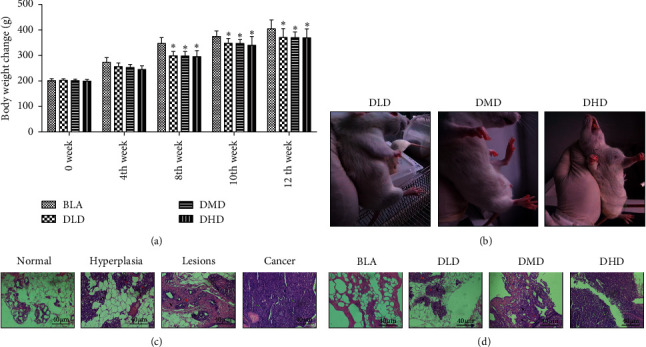
General condition, weight change, and breast tissue morphological change in the breast precancerous lesion rat model. (a) Body weight change of rats in each group. (b) The picture of visible breast tumors of rats in each group. (c) Rats' breast tissue morphological detection in the process from normal, hyperplasia, and lesions to cancer. (d) Breast tissue morphological assay for breast precancerous lesion model rats at the 10th week. Data were expressed as means ± standard deviation. ^*∗*^*P* < 0.05 compared with the BLA group. The black arrows represent inflammation, the red arrows represent hyperplasia, and the green arrows represent carcinoma.

In order to identify the optimal dose and time for inducing the breast precancerous lesion in the rat model, different doses of DMBA-combined estrogen and progesterone were used to treat the rats for 8, 10, and 12 weeks. Results of body weight change are summarized in Figure 1(a). The gain in body weight was significantly less in the DLD, DMD, and DHD groups compared with the BLA group after DMBA induced more than 8 weeks.

After DMBA administration, some rats developed mild diarrhea, which gradually disappeared after 2–4 weeks. The rats in the BLA group had good hair color, agile activities, and no obvious enlargement of the mammary gland. The rats in each model group showed dark hair color, slow reaction, and irritability after DMBA induced more than 4 weeks (data not shown). From the 8th week, tumors were visible in each model group, and the number of tumors in the DMD and DHD groups was significantly higher than that in the DLD group (Table 1 and Figure 1(b)). Morphological changes in the breast were evaluated with H&E staining (Figures 1(c) and 1(d)). The precancerous lesion and invasive carcinoma did not occur in the BLA group at 8th, 10th, and 12th week. But, varying degrees of precancerous lesions and invasive carcinomas occurred in the DLD, DMD, and DHD groups, indicating that DMBA-combined estrogen and progesterone can successfully induce the breast precancerous lesion in rats. At the 12th week, 25% of the rats in the DMD group and 41.7% in the DHD group had developed breast cancer, missing the best opportunity for a precancerous lesion model. However, the incidence of precancerous lesions in the DMD group reached 91.7% at the 10th week (Table 2). Thus, 10 mg/each rat of DMBA and 10 weeks were relatively an ideal dose and time for inducing the breast precancerous lesion rat model.

### 3.2. XTJY Had Effective Therapeutic Intervention Effects on Breast Precancerous Lesion Rats

**Figure 2:**
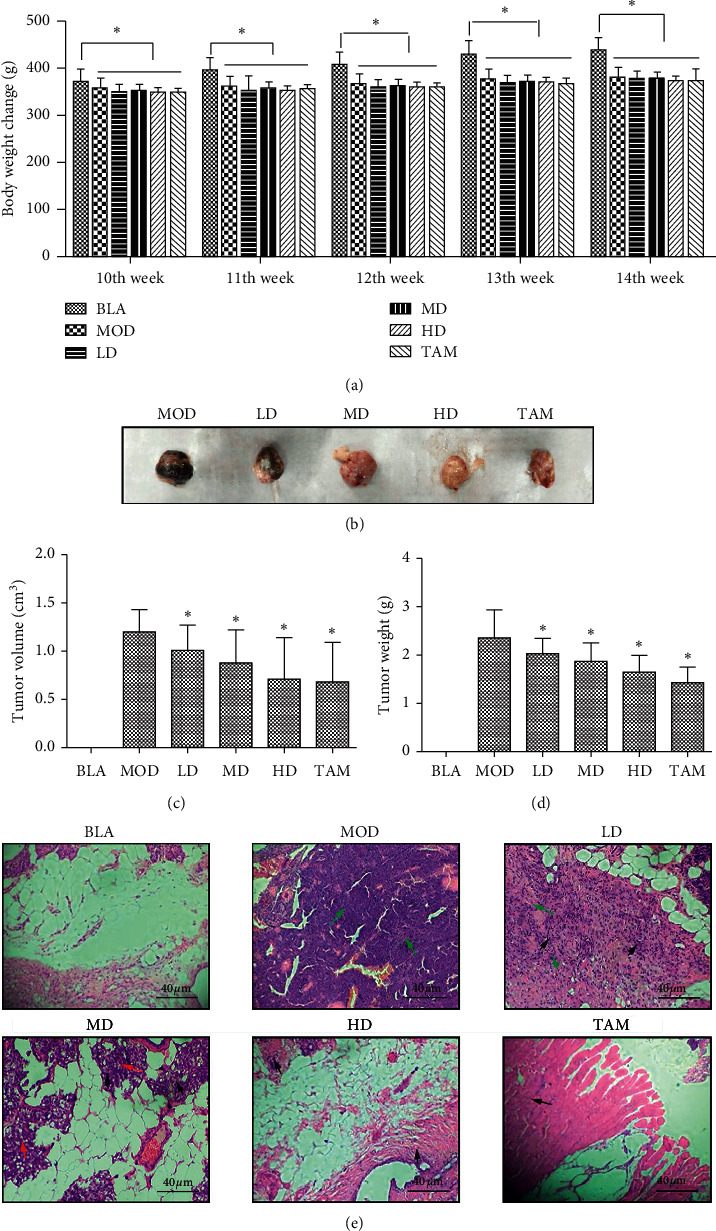
Weight change and breast tissue morphological change in the breast precancerous lesion rat model after TAM or XTJY treatment for 4 weeks. (a) Body weight change of rats in each group. (b) Representative tumors were photographed for 4 weeks after the first treatment with TAM or XTJY. (c) Mean tumor volumes in each group. (d) Mean tumor weight in each group. (e) Breast tissue morphological assay for breast precancerous lesion model rats after TAM or XTJY treatment for 4 weeks. Data were expressed as means ± standard deviation. ^*∗*^*P* < 0.05 compared with the BLA group. The black arrows represent inflammation, the red arrows represent hyperplasia, and the green arrows represent carcinoma.

Rats in the LD, MD, and HD groups were treated with XTJY of different concentrations to assess the therapeutic effect of XTJY on breast precancerous lesion rats. Results of body weight change after XTJY or TAM treatment are summarized in Figure 2(a). The gain in body weight was significantly less in the MOD, LD, MD, HD, and TAM groups compared with the BLA group. The rats in the BLA group exhibited weight loss, dull skin, and dramatically reduced activity. After treated with XTJY or TAM from the 11th week, these symptoms improved, and dietary behavior was basically normal in the breast precancerous lesion rats compared with the MOD group (data not shown). After treatment for 4 weeks, tumors were visible in each group except for the BLA group, and the number of tumors in the treatment group was significantly lower than that in the MOD group (Table 3), indicating that XTJY or TAM treatment can effectively reduce the incidence of visible tumors, and XTJY prescription showed dose-dependent in the process of inhibiting the breast precancerous lesion. In addition, tumor volume and tumor weight in the treatment group were significantly decreased compared with the MOD group (Figures 2(b)–2(d)), indicating that XTJY can inhibit breast tumor cell proliferation in a dose-dependent manner. However, there were no obvious changes in the thymus index and spleen index in each group (Table 4). Pathomorphological observation on the breast tissues of rats showed that no precancerous lesion and invasive carcinoma were found in the BLA group, and the atypical hyperplasia rate was 10%, while the precancerous lesion rate was 30% and the invasive carcinoma rate was 70% in the MOD group. After intervention by TAM or XTJY prescription at different doses, the rate of precancerous lesion and invasive carcinoma was lower than that in the MOD group, among which the invasive carcinoma of rats in the HD and TAM groups was reduced to 20% (Figure 2(e) and Table 5). These results indicated that TAM or XTJY prescription can effectively reduce the incidence of breast invasive carcinoma in rats, and the treatment effect of high-dose XTJY and TAM is equivalent.

### 3.3. XTJY Treatment Inhibits the Activation of the PI3K/Akt Signaling Pathway In Vivo

**Figure 3:**
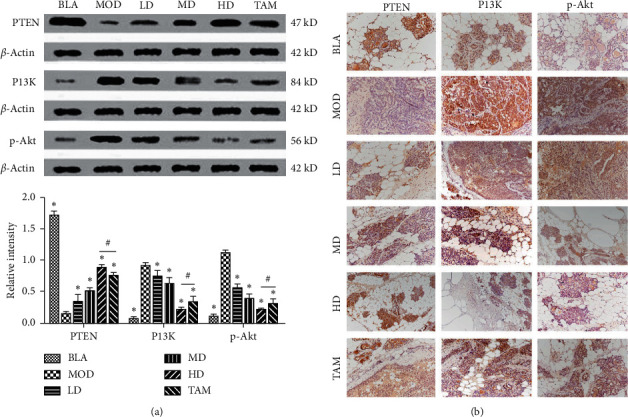
The expression of PTEN, PI3K, and p-Akt in the breast precancerous lesion rat model after TAM or XTJY treatment for 4 weeks. (a) The protein expression levels of PTEN, PI3K, and p-Akt were examined by western blot assay. (b) Immunohistochemical assay was performed to detect the expression levels of PTEN, PI3K, and p-Akt in breast tissues. Data were expressed as means ± standard deviation. ^*∗*^*P* < 0.05 compared with the MOD group; ^#^*P* < 0.05.

The effects of XTJY on the expression of PI3K/Akt signaling pathway key proteins were detected using a western blot assay. As shown in Figure 3(a), compared with the MOD group, the expression levels of PI3K and p-Akt were significantly decreased in other groups, while the expression level of PTEN was significantly increased in other groups. Compared with the TAM group, the expression levels of PI3K and p-Akt were significantly reduced in the HD group, while the expression level of PTEN was significantly increased in the HD group, indicating that the inhibition effect of high-dose XTJY on the PI3K/Akt signaling pathway was stronger than TAM. As shown in Figure 3(b), the immunohistochemical staining results revealed that PTEN and PI3K were mainly expressed in the cytoplasm and cell membrane, and p-Akt protein was expressed in the nucleus. Compared with the MOD group, the expression of PTEN in the treatment group was enhanced to varying degrees, showing that the positive brown-yellow area was enlarged, among which the protein expression of PTEN in the high-dose XTJY group was the strongest. Compared with the MOD group, the expression of PI3K and p-Akt in the treatment group was decreased to varying degrees, showing that the positive brown-yellow area is becoming smaller and the color becoming lighter, among which the expression of PI3K and p-Akt in the high-dose XTJY group was the most obvious. These data indicated that the PI3K/Akt signaling pathway has been activated in the breast precancerous lesion, and XTJY treatment can inhibit the activation of the PI3K/Akt signaling pathway so as to delay or reverse precancerous lesions.

## 4. Discussion

Breast cancer is the most common invasive cancer in women worldwide, and it imposes a heavy burden on patients [11]. Clinically, patients with benign breast disease are under a significantly higher risk of breast cancer than healthy people [12]. Carcinogenesis is a gradual and continuous process, and the process before the occurrence of carcinoma in situ is reversible. Precancerous lesion is an important step of breast cancer progression. Clinically, tamoxifen is mainly used for the treatment of breast precancerous lesion and the early prevention of breast cancer. However, tamoxifen is prone to cause endocrine disorders and liver and kidney damage [13]. Chinese materia medica (CMM) is the typical “multicomponent, multitarget, and multipathway” agent. In recent years, multiple TCM compound prescription has been reported to play an important role in antitumor [14,15], including antibreast cancer [16,17]. However, there are limited reports on the treatment of precancerous breast cancer with the TCM compound prescription based on TCM theory. The present study aimed to investigate the therapeutic effect and the possible mechanism of XTJY on breast precancerous lesions in vivo. It demonstrated that XTJY had obvious therapeutic effects on breast precancerous lesions by blocking the PI3K/Akt signaling pathway.

In order to make an animal model of breast precancerous lesion which corresponds with the demand of the research, the models must simulate a continuous process containing normal, hyperplasia, atypical hyperplasia, and carcinoma [18]. Currently, DMBA-combined estrogen and progesterone-induced breast precancerous lesion is the most common method for studying breast precancerous lesions in animal models [19]. However, the dose and time of DMBA-induced breast precancerous lesions in rats are different. Min et al. [10] reported that 7 mg/each rat DMBA-combined estrogen and progesterone induced for 60 d, the incidence of premalignant breast disease was 87.5%. Research of Yang [20] showed that the rats received one-time gavage using 5 mg/each rat DMBA and were conventionally fed for 90 d, and the incidence of breast hyperplasia was 75%. In the present study, the DMBA of 5 mg, 10 mg, or 20 mg/each rat combined with estrogen and progesterone were used to treat the rats for 8, 10, and 12 weeks. It demonstrated that the detection rate of breast precancerous lesions in rats was the highest after 10 mg/each rat DMBA combined with estrogen and progesterone induced for 10 weeks. Pathological results revealed that the whole pathological process of breast tissue from normal to hyperplasia, atypical hyperplasia, and carcinoma was presented in the precancerous lesion rat model, indicating that the model could be used to study the mechanism of precancerous lesion and evaluate the drug efficacy.

For breast precancerous lesions, professor Pin-Kang Wei believes that the poison of phlegm-blood stasis and the depression of Qi are the basic pathogenesis of breast precancerous lesions. “XTJY prescription” is created by professor Pin-Kang Wei after summarizing years of clinical experience. In the prescription, rhizoma arisaematis, *Pinellia ternata*, and Indian Iphigenia Bulb were the main drugs to eliminate phlegm; *Bupleurum*, fried *Atractylodes*, *Angelica sinensis*, and hangbaishao are complementary drugs for relieving depression; and peppermint as adjuvant can regulate Qi function. Above with the use of herbs is mainly to dissolve phlegm and detoxify, as well as to regulate Qi depression [9]. Clinical practice has proved that XTJY prescription can effectively treat breast precancerous lesions [21]. In addition, our previous study also demonstrated that XTJY can induce apoptosis of human breast precancerous lesion MCF-10AT cells [9]. However, there is no evidence to further verify that XTJY has an effective intervention effect on breast precancerous lesions in vivo. In the present study, it demonstrated that XTJY prescription can not only improve the general physiological conditions, but also improve the mood of rats with breast precancerous lesions. The pathological diagnosis results revealed that XTJY prescription can significantly reduce the incidence of breast cancer in the precancerous lesion rats. These data indicated that XTJY prescription had good therapeutic effect on breast precancerous lesions in rats.

As a critical regulator of apoptosis, the PI3K/Akt signaling pathway regulates important biological processes including tumorigenesis [22, 23]. The PI3K/Akt signaling pathway has been shown to be over-activated in breast cancer [24, 25]. Therefore, inhibiting the activation of PI3K/Akt signaling pathway may be the main mechanism for the treatment of breast cancer. Zhang et al. [25] reported that astragaloside IV treated breast cancer through up-regulation of Nrf2 via inhibiting PI3K/AKT/mTOR signaling pathway. Fedele et al. [26] reported that INPP4B functions as a tumor suppressor by negatively regulating normal and malignant mammary epithelial cell proliferation through inhibition of the PI3K/Akt signaling pathway. In addition, eugenol alleviated breast precancerous lesions through HER2/PI3K-AKT pathway-induced cell apoptosis and S-phase arrest. In the present study, the XTJY treatment group exhibited significantly higher protein expression of PTEN and lower protein expression of PI3K and p-Akt, indicating that XTJY alleviated breast precancerous lesions of rats through inhibiting the activation of the PI3K/Akt signaling pathway. However, this study is not without limitations. In order to further clarify the role of the PI3K/Akt signaling pathway in the inhibition of XTJY prescription in breast precancerous lesions, we will construct gene knockout rat in the next stage and reveal the therapeutic target of XTJY prescription in the treatment of breast precancerous lesions

## 5. Conclusion

In summary, the present study suggests that XTJY can effectively alleviate the development of breast precancerous lesions through inhibiting the PI3K/Akt signaling pathway, indicating that XTJY may be a promising TCM prescription for the prevention or treatment of breast precancerous lesions. This study provides an experimental basis for the further application of XTJY prescription in clinical treatment of breast precancerous lesions.

## Figures and Tables

**Table 1:** Visible breast tumors of rats in each group.

Group	The number of breast tumors of rats			
	4th week	8th week	10th week	12th week
BLA (*n* = 5)	0/5	0/5	0/5	0/5
DLD (*n* = 12)	0/12	3/12 (25.0%)^*∗*^	5/12 (41.7%)^*∗*^	7/12 (58.3%)^*∗*^
DMD (*n* = 12)	0/12	7/12 (58.3%)^*∗*^	11/12 (91.7%)^*∗*^	12/12 (100%)^*∗*^
DHD (*n* = 12)	0/12	9/12 (75.0%)^*∗*^	11/12 (91.7%)^*∗*^	12/12 (100%)^*∗*^

Data were expressed as means ± standard deviation. ^*∗*^*P* < 0.05 compared with the BLA group.

**Table 2:** Results of breast tissue pathological morphology of rats in each group at 8th, 10th, and 12th week.

Group	Normal	Hyperplasia	Lesions	Cancer
8th week				
BLA (*n* = 5)	5 (100%)	0	0	0
DLD (*n* = 12)	0	9 (75.0%)	3 (25.0%)	0
DMD (*n* = 12)	0	5 (41.7%)	7 (58.3%)	0
DHD (*n* = 12)	0	3 (25.0%)	8 (66.7%)	1 (8.3%)

10th week				
BLA (*n* = 5)	5 (100%)	0	0	0
DLD (*n* = 12)	0	7 (58.3%)	5 (41.7%)	0
DMD (*n* = 12)	0	1 (8.3%)	11 (91.7%)	0
DHD (*n* = 12)	0	1 (8.3%)	8 (66.7%)	3 (25.0%)

12th week				
BLA (*n* = 5)	5 (100%)	0	0	0
DLD (*n* = 12)	0	5 (41.7%)	6 (50.0%)	1 (8.3%)
DMD (*n* = 12)	0	0	9 (75.0%)	3 (25.0%)
DHD (*n* = 12)	0	0	7 (58.3%)	5 (41.7%)

**Table 3:** The number of visible breast tumors in each group after treatment for 4 weeks.

Groups	Number of tumors	Incidence rate
BLA (*n* = 20)	0	0
MOD (*n* = 20)	18	90%
LD (*n* = 19)	14	73.7%
MD (*n* = 19)	12	63.2%
HD (*n* = 20)	9	45.0%
TAM (*n* = 20)	10	50.0%

**Table 4:** Thymus index and spleen index in rats in each group after treatment for 4 weeks.

Group	Thymus index	Spleen index
BLA (*n* = 20)	0.36 ± 0.24	0.67 ± 0.12
MOD (*n* = 20)	0.31 ± 0.13	0.64 ± 0.08
LD (*n* = 19)	0.32 ± 0.18	0.64 ± 0.17
MD (*n* = 19)	0.31 ± 0.10	0.68 ± 0.28
HD (*n* = 20)	0.32 ± 0.19	0.68 ± 0.19
TAM (*n* = 20)	0.32 ± 0.16	0.67 ± 0.08

**Table 5:** Results of breast tissue pathological morphology of rats in each group after treatment for 4 weeks.

Group	Normal		Hyperplasia		Lesions		Cancer	
BLA (*n* = 20)	18	90%	2	10.0%	0	0	0	0
MOD (*n* = 20)	0	0	0	0	6	30%	14	70%
LD (*n* = 19)	0	0	0	0	8	44.4%	11	57.9%
MD (*n* = 19)	0	0	2	10.5%	10	52.6%	7	36.8%
HD (*n* = 20)	0	0	5	25%	11	55%	4	20%
TAM (*n* = 20)	0	0	4	20%	12	60%	4	20%

## Data Availability

The datasets used or analyzed during the current study are available from the corresponding author on reasonable request.
